# Efavirenz treatment improves retinal vaso-obliteration and pathological neovascularization in a mouse model of retinopathy of prematurity

**DOI:** 10.3389/fmed.2026.1745351

**Published:** 2026-02-23

**Authors:** Briah Bailey, Josephine Rudd Zhong Manis, Gayatri Seth, Shubhra Rajpurohit, Allston Oxenrider, Pamela M. Martin, Ravirajsinh N. Jadeja, Menaka C. Thounaojam

**Affiliations:** 1 Department of Biomedical Sciences, School of Graduate Studies, Meharry Medical College, Nashville, TN, United States; 2 Department of Cellular Biology and Anatomy, Medical College of Georgia at Augusta University, Augusta, GA, United States; 3 Department of Ophthalmology, Medical College of Georgia at Augusta University, Augusta, GA, United States

**Keywords:** cholesterol metabolism, CYP46A1, efavirenz, prematurity, retinopathy

## Abstract

**Objectives:**

Previous studies have shown the metabolic and regulatory significance of CYP46A1 in the adult retina; however, its role in the developing retina is unknown. Here, we evaluate CYP46A1 expression and the impact of its activation in the developing mouse retina under normal and pathological conditions.

**Methods:**

Seven-day-old (P7) C57BL/6 J mice maintained in room air (controls) or subjected to oxygen-induced retinopathy (OIR) were treated with/without 20 mg/kg efavirenz (EFV), a CYP46A1 activator administered intraperitoneally from P7 to P17.

**Results:**

Retinal cross sections and flat mounts were prepared to study retinal vasculature morphology, Müller and microglia activation, and ganglion cell viability. EFV treatment significantly reduced pathological neovascularization and the size of avascular and hypoxic areas in OIR mice retinas. EFV treatment additionally limited reactive gliosis and microglia activation and improved retinal ganglion cell survival in OIR mice.

**Conclusion:**

The current study demonstrates the developmental regulation of CYP46A1 and the dysregulated expression and levels of the downstream metabolite 24-Hydroxycholesterol (24HC) in OIR mice. The study further suggests that EFV treatment (in part via CYP46A1 activation) may improve key pathological features associated with pathological neovascularization in OIR mice.

## 1 Introduction

Retinopathy of prematurity (ROP), an ocular complication of prematurity, is a leading cause of childhood blindness (1–3). Low birth weight (≤1,500 g), low gestational age (≤30 weeks), and prolonged oxygen therapy are consistently associated with increased disease risk (1–3). To date, therapies for ROP are applied in the most advanced, proliferative stages of the disease and essentially involve ablation of the peripheral retina through surgical procedures like laser photocoagulation, cryotherapy, and vitrectomy (4–6). Intravitreal injections of anti-vascular endothelial growth factor (VEGF) are also commonly applied to halt retinal neovascularization (RNV) (4–6). However, this treatment may also block the normal vascular development still taking place in the premature infant retina. Therefore, even when treated, children affected by ROP may still experience substantial vision loss and/or are more likely than others to develop visual impairments later in life (e.g., nearsightedness, strabismus, amblyopia, and glaucoma) (7–10). Therefore, identifying novel mechanisms and developing new therapies for ROP is essential.

Bile acids have significant potential with respect to limiting RNV (11–13). However, the underlying mechanisms are not fully understood. Bile acids are essential for cholesterol catabolism, and cytochrome P450 (CYP) enzymes are critically involved. Several studies have implicated roles for CYP enzymes in the regulation of retinal angiogenesis (14, 15). However, whether they are expressed and function similarly in the premature retina is not known. In preterm infants with ROP, enzymes regulating cholesterol and fatty acid metabolism-CYP1B1, CYP2C8, COX2, and ALOX15-are markedly upregulated, indicating lipid dysregulation [ref]. Furthermore, experimental studies show that ACAT1-mediated inhibition of cholesterol esterification significantly reduces retinal neovascularization independently of VEGF expression [36691048], highlighting the significance of cholesterol metabolism in the developing retina. The present study investigates the expression of CYP27A1 and CYP46A1 in the developing mammalian retina in the context of pathologic angiogenesis using oxygen-induced retinopathy (OIR) mice. CYP27A1 functions as a sterol 27-hydroxylase, whereas CYP46A1 is responsible for cholesterol 24-hydroxylation (16, 17). Both CYPs play crucial roles in removing cholesterol by generating 24-hydroxycholesterol (24HC) and 27-hydroxycholesterol (27HC), oxysterols that facilitate the movement of tissue cholesterol to the systemic circulation (18–20). 24HC and 27HC also interact with liver X receptors (LXRs) (21), and serve as substrates for the synthesis of primary bile acids in the retina (22), which is important given the protective signaling elicited by bile acids in retinal diseases, including ROP (13).

The current study is the first to demonstrate CYP27A1 and CYP46A1 in the normal developing mammalian retina. Further, our findings expose a novel regulatory role for CYP46A1 in the pathologic angiogenesis characteristic of OIR/ROP and establish CYP46A1 as a therapeutic target for mitigating retinal inflammation and neovascularization for improved ROP management.

## 2 Materials and methods

### 2.1 Oxygen-induced retinopathy (OIR) model

All animal procedures adhered to Association for Research in Vision and Ophthalmology guidelines and were approved by the Meharry Medical College Institutional Animal Care and Use Committee. C57BL/6 J mice (Jackson Laboratories, Bar Harbor, ME, USA) were housed under standard conditions with a 12-h light/dark cycle and ad libitum access to food and water.

OIR was induced following the Smith et al. protocol, previously validated in our studies (11, 23, 24). On postnatal day 7 (P7), pups and their dam were placed in a hyperbaric chamber (BioSpherix, Parish, NY, USA) with iris ports enabling *in vivo* manipulation without disrupting oxygen levels. Oxygen was maintained at 75% from P7 to P12 to induce central vaso-obliteration. Mice were then returned to room air (21% oxygen) until P17 to promote retinal neovascularization (RNV). Age-matched controls were kept in room air from P0 to P17.

### 2.2 Efavirenz (EFV) administration

EFV (Cayman Chemical, Ann Arbor, MI, USA) was dissolved in 0.01% DMSO and administered intraperitoneally at 20 mg/kg/day from P7 to P17. The OIR control mice (OIR group) received 0.01% DMSO alone and served as a vehicle control for the OIR + EFV group. Eyes were harvested at P17 for downstream analyses.

### 2.3 Retinal vascular quantification

At P17, eyes were fixed in 4% paraformaldehyde, and retinas dissected for flat mount preparation. Vessels were stained using biotinylated Isolectin GS-IB4 (0.2 mg/mL; Invitrogen, Carlsbad, CA, USA) and Texas Red–conjugated avidin D overnight at 4 °C. Imaging was performed using a Zeiss LSM 780 Inverted Confocal microscope. Quantification of vaso-obliteration and neovascularization was automated using the OIRSeg tool.^1^ Angiogenesis parameters were further analyzed using AngioTool software (NIH/NCI).

### 2.4 Western blot analysis

Retinal lysates were prepared at P7, P12, P14, P17, and P23 using RIPA buffer (Thermo Fisher, Waltham, MA, USA) supplemented with 1% phosphatase and protease inhibitors (Sigma-Aldrich, St. Louis, MO, USA). Protein (30–50 μg) was resolved by SDS-PAGE and transferred to PVDF membranes. Membranes were blocked with 5% skim milk and probed with primary antibodies: CYP46A1 (1:1000, RayBiotech), CYP27A1 (1:1000, Abcam), RBPMS (1:500, GeneTex), VCAM-1 (Abclonal), and ICAM-1 (1:1000, Santa Cruz Biotechnology). *β*-actin (1:3000, Sigma-Aldrich) was used as a loading control. Secondary detection was performed using chemiluminescence (Thermo Fisher) and Azure 300 Imaging System. Band intensities were quantified using NIH ImageJ software and expressed as fold change relative to controls.

### 2.5 Immunofluorescence

Retinal cryosections and flat mounts were fixed in 4% paraformaldehyde, blocked with 1X Power Block (Biogenex), and incubated overnight at 4 °C with primary antibodies: RBPMS (1:500, GeneTex), GFAP (1:100, Abcam), and IBA-1 (1:100, FUJIFILM Wako). After washing with 0.1% Triton X-100 in PBS (pH 7.4), slides were incubated with fluorescent secondary antibodies (Life Technologies) and mounted with DAPI-containing fluoroshield (Sigma-Aldrich). Images were acquired at 20X magnification using a Zeiss Axioplan-2 fluorescence microscope. All images were captured from three adjacent fields on both the temporal and nasal sides of the optic nerve, providing a standardized approach for analysis. RBPMS-positive ganglion cells and GFAP projections were counted and averaged across both regions per eye. Three eyes per group were quantified. For IBA-1 quantification, the numbers of Quiescent and ramified cells were counted in 4 regions of each retinal flat mount, averaged, and expressed as a ratio across 3 independent retinal flat mounts.

### 2.6 RNA sequencing

Total RNA was extracted from RA17 and OIR17 retinas using the RNeasy kit (Qiagen). cDNA libraries were generated via strand-specific amplification and sequenced using paired-end 50-bp reads on an Illumina HiSeq 2,500 platform at Augusta University’s Genomics Core. Data were analyzed using Tophat2 and Cufflinks, and heatmaps were generated to visualize differential gene expression in sterol metabolism pathways.

### 2.7 ELISA for 24S-hydroxycholesterol

Retinal homogenates from RA, OIR, and EFV-treated OIR mice were analyzed using the 24S-Hydroxycholesterol ELISA Kit (Abcam, ab204530). Samples were incubated in wells pre-coated with goat anti-rabbit IgG, followed by biotinylated 24HC and streptavidin-HRP. After the addition of TMB substrate, absorbance was measured at 450 nm. Concentrations were calculated from a standard curve and expressed as ng/g retina.

### 2.8 Cell culture

Human microglial cells (HMC3; ATCC® CRL-3304™) were maintained in Eagle’s Minimum Essential Medium (EMEM) supplemented with 10% fetal bovine serum (FBS) and 1% penicillin–streptomycin. Cultures were incubated at 37 °C in a humidified atmosphere containing 5% CO₂.

Human retinal pigment epithelial cells (ARPE-19; ATCC® CRL-2302™) were maintained in Dulbecco’s Modified Eagle Medium/Nutrient Mixture F-12 (DMEM/F12) supplemented with 10% fetal bovine serum (FBS) and 1% penicillin–streptomycin. Cultures were incubated at 37 °C with 5% CO₂ in a humidified atmosphere.

Primary HRMECs (ACBRI-181; Cell Systems) were maintained in Complete Classic Medium with CultureBoost™ (Cell Systems) or equivalent endothelial growth medium supplemented with 10% fetal bovine serum (FBS), growth factors, and antibiotics (penicillin–streptomycin). Cultures were incubated at 37 °C in a humidified atmosphere with 5% CO₂.

Primary human retinal astrocytes (ScienCell Research Laboratories) were maintained in Astrocyte Medium (AM; ScienCell) supplemented with 2% fetal bovine serum (FBS), 1% astrocyte growth supplement (AGS), and 1% penicillin–streptomycin. Cultures were incubated at 37 °C in a humidified atmosphere with 5% CO₂.

R28 retinal neuronal-like cells (Kerafast, Inc., Boston, MA) were cultured in DMEM with low glucose (Sigma Aldrich) supplemented with 10% fetal calf serum (Cytiva HyClone) and 1% penicillin–streptomycin (Gibco), 7.5% sodium bicarbonate (Sigma, cat# S8761), 1% MEM non-essential amino acids (Gibco). R28 cells were differentiated overnight in a complete medium supplemented with 250 μM pCPT-cAMP (Sigma Aldrich) on laminin-coated plates. Cells were then processed for the experiments as required.

### 2.9 Statistical analysis

Data are presented as mean ± SEM from 3–7 biological replicates. Statistical analyses were performed using the Kruskal–Wallis test for multiple group comparisons. If a significant difference was found, *post hoc* pairwise comparisons were carried out using Dunn’s multiple-comparison test with adjusted *p*-values. Unpaired t-tests were used for planned pairwise comparisons between two groups where appropriate. Statistical significance was set at *p* < 0.05. Graphs were generated using GraphPad Prism v10 (GraphPad Software, San Diego, CA, USA).

## 3 Results and discussion

Retina intricately balances cholesterol synthesis and output pathways to avoid the detrimental effects of excess tissue cholesterol (25, 26). Cholesterol conversion to oxysterols (27-hydroxycholesterol, 27HC; 5-cholestenoic acid, 27COOH; 7α-hydroxy-3-oxo-4-cholestenoic acid, 7HCA; and 24-hydroxycholesterol, 24HC) by cytochrome P450 enzymes 27A1 and 46A1 (CYP27A1 and CYP46A1, respectively) is key to elimination (25, 26). Further, 27HC and 24HC interact with LXR (27) and serve as substrates for the synthesis of primary bile acids in the retina. The importance of CYP27A1 and CYP46A1 in the retina is highlighted further by the demonstration of defective cholesterol clearance/increased lipid deposition and retinal neovascularization in adult mice lacking CYP27A1 and CYP46A1 (16, 27). However, the expression and function of these enzymes in the developing retina and ROP remain unknown.

**Figure 1:**
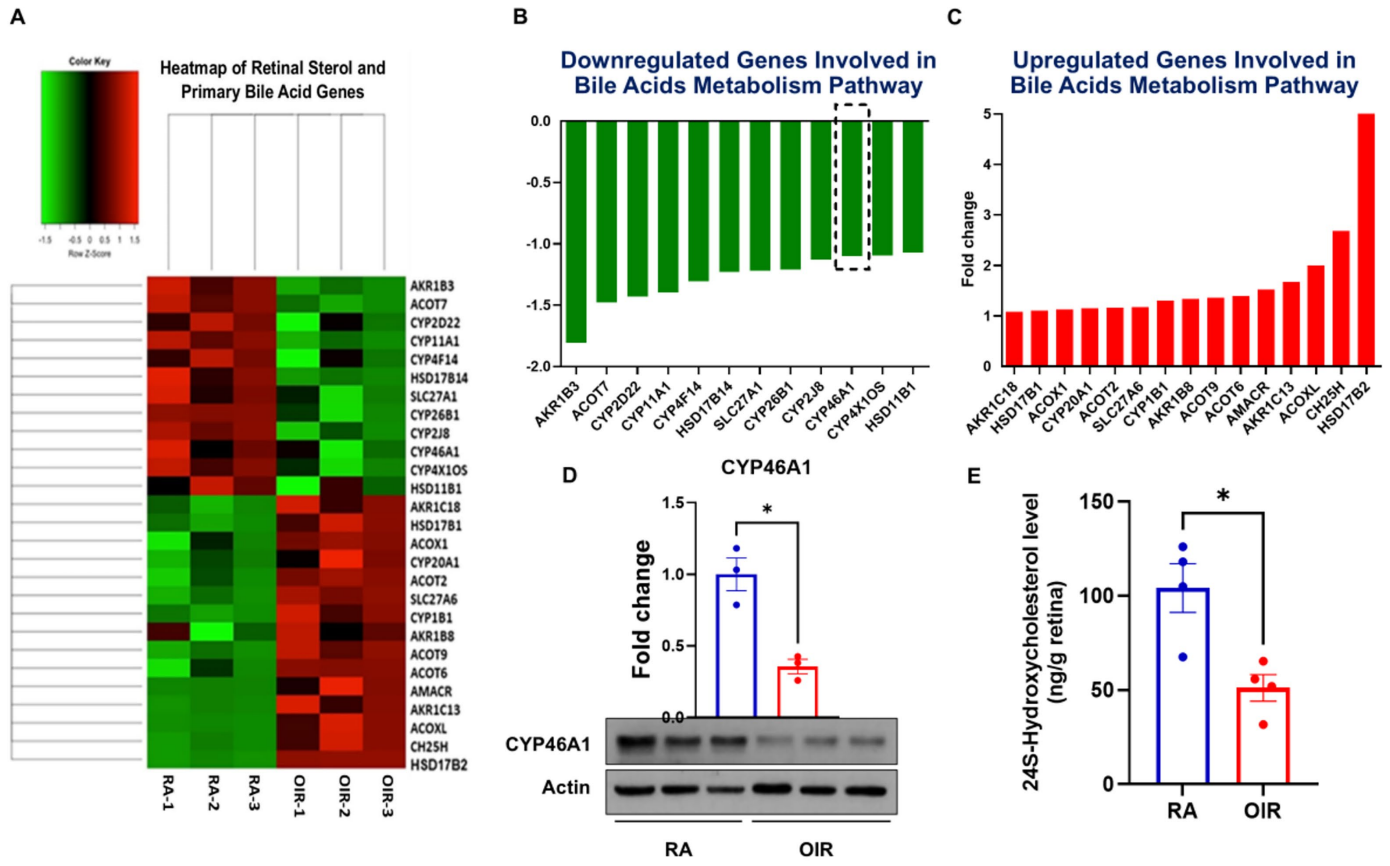
RNA sequencing was performed using total RNA extracted from room air (RA) and oxygen-induced retinopathy (OIR) mouse retinas at postnatal day 17 (P17). **(A)** Heat map illustrating genes up- (red) or down-regulated (green) in sterol metabolism pathways, expressed as fold-changes in gene expression in panels **(B,C)**. Results are shown as mean for *n* = 3 retinas per group; each obtained from a different mouse. **(D)** Western blot evaluation of CYP46A1 protein expression in RA and OIR mice. Values are presented as mean ± SEM (*n* = 3 retinas per group, each obtained from a different mouse). **(E)** ELISA quantification of 24S-hydroxycholesterol in retinal samples showing changes in 24S-Hydroxycholesterol levels in OIR mice. Values are presented as mean ± SEM (*n* = 3–4 retinas per group, each obtained from a different mouse). *p < 0.05 vs. RA or OIR.

**Figure 2:**
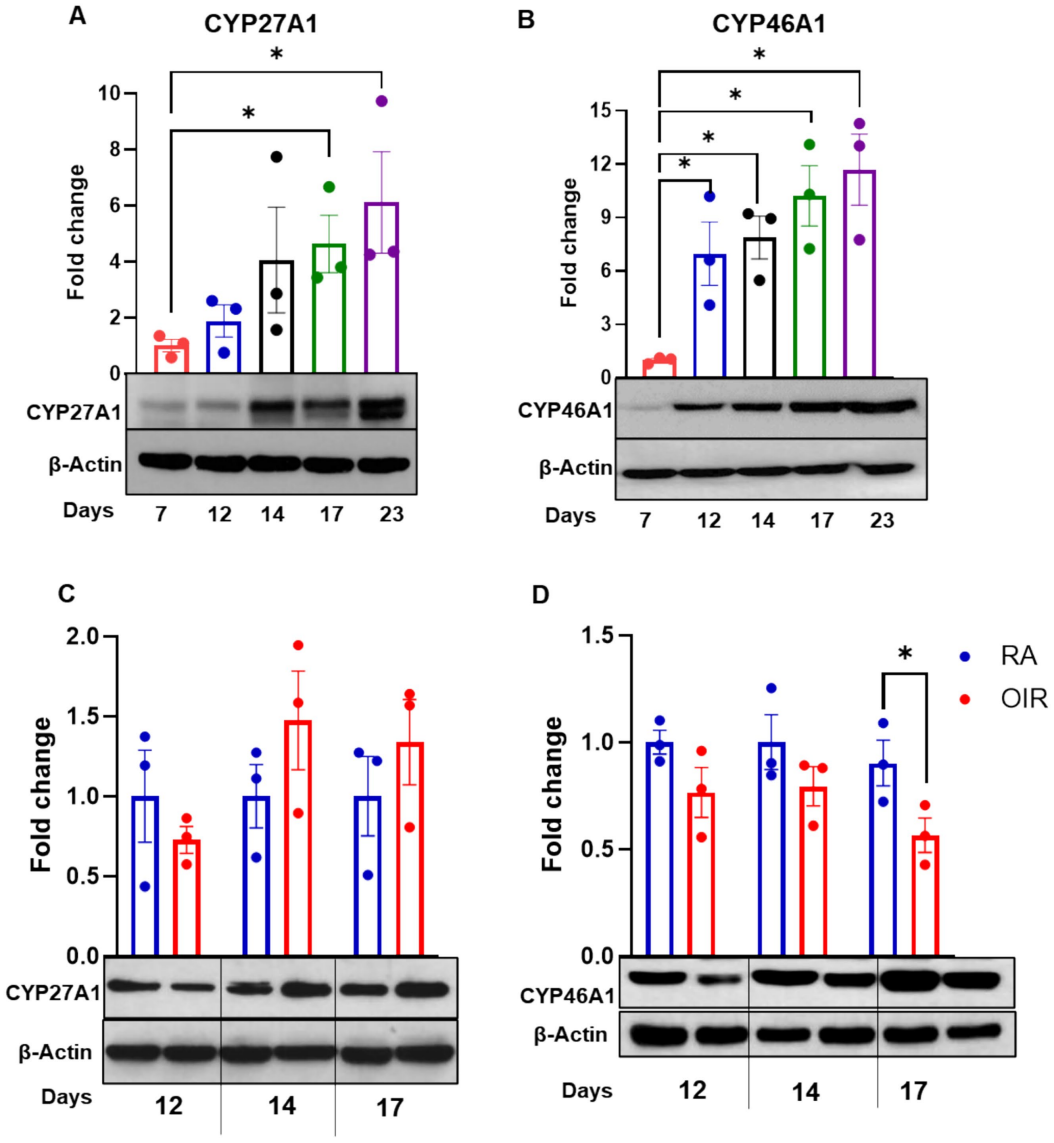
**(A,B)** Western blot evaluation of CYP27A1 and CYP46A1 protein in the developing mouse retina (postnatal day 7 to 17), or **(C,D)** in mice maintained in room air (RA) or subjected to oxygen-induced retinopathy (OIR) at P12, 14, and 17. Results were normalized to *β*-actin and expressed as fold change; mean ± SEM (*n* = 3 retinas per group, each obtained from a different mouse). *p < 0.05 vs. postnatal day 7 (P7) or RA.

The mammalian retina continues to develop postnatally. Understanding key metabolic and molecular changes that occur pre- and postnatally in health and disease, the purpose of the present study, may provide critical information toward the development of new therapies for neonatal retinal diseases. We and others have demonstrated the therapeutic potential of bile acids in retinal neovascularization (11, 13, 22). However, the underlying mechanisms to explain these effects are not fully understood. RNA sequencing analyses (Figures 1A–C) performed using retinal samples obtained from RA and OIR mice at P17 confirmed the alteration of key players in sterol and BA synthesis pathways in OIR mice. CYP46A1 was notably downregulated compared to RA controls (Figure 1B), as confirmed by Western blotting of CYP46A1 expression (Figure 1D) and ELISA analyses for levels of 24HC (Figure 1E). Interestingly, given the surrogate relationship between CYP27A1 and CYP46A1 (19), RNA sequencing studies did not reveal significant alterations in CYP27A1. However, because little is known regarding the expression and functional relevance of these enzymes in the normally developing retina, we evaluated their expression in P7-P23 mouse retinas. The expression of both CYPs was evident by P12 and increased progressively with age (Figures 2A,B). However, congruent with RNA sequencing data above, only CYP46A1 expression was disrupted significantly in OIR (Figures 2C,D). As such, the remainder of the study focuses on CYP46A1 expression and function in OIR.

**Figure 3:**
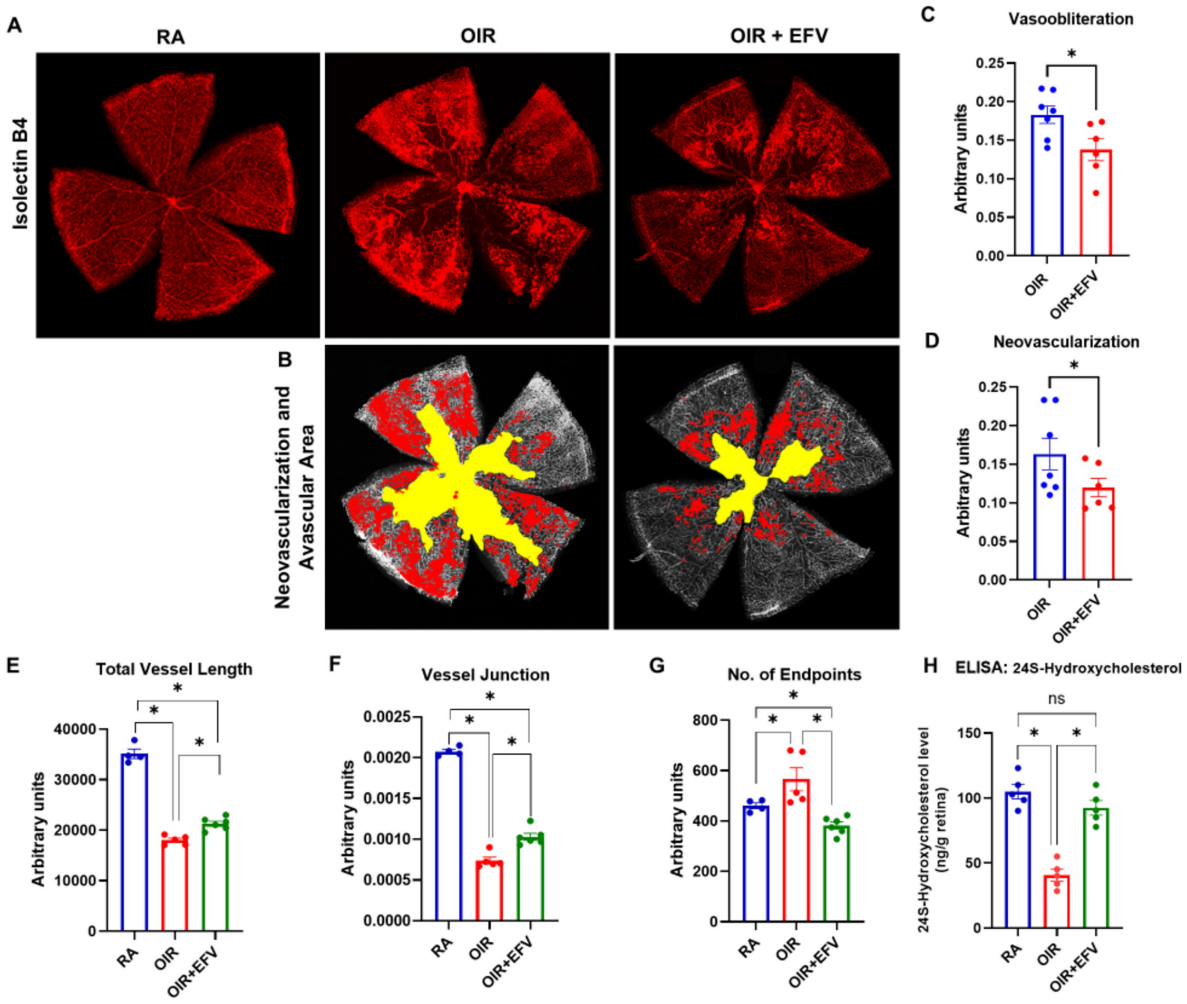
**(A)** Representative isolectin B4-stained retinal flat mounts prepared from room air (RA), oxygen-induced retinopathy (OIR), and efavirenz (EFV)-treated OIR (20 mg/kg; P7–P17) sacrificed at P17. **(B)** Representative retinal flat mounts in which the area of neovascularization (red) and avascular area (yellow) have been highlighted. Retinal images were further processed using OIRSeg, an unbiased automated web application, to quantify changes in **(C)** vaso-obliteration **(D)** neovascularization. Further, vessel length **(E)**, vessel junctions **(F)**, and number of endpoints **(G)** were calculated using AngioTool analysis. Values are presented as mean ± SEM (*n* = 5–7 retinas per group, each obtained from a different mouse). *p < 0.05 vs. RA or OIR. **(H)** An ELISA was performed on retinal samples to compare levels of 24S-Hydroxycholesterol after activation of CYP46A1 by EFV treatment. Values are presented as mean ± SEM (n = 5 retinas per group). *p < 0.05 vs. RA or OIR.

Because CYP46A1 expression was found to be downregulated in OIR, we next evaluated the impact of CYP46A1 expression in OIR pathology in the presence or absence of the CYP46A1 activator efavirenz (EFV). EFV, an FDA-approved antiretroviral medication for HIV treatment, has also been tested in clinical trials in patients with mild cognitive impairment or early dementia due to Alzheimer’s disease (28). EFV activates CYP46A1 by binding to the allosteric site on the CYP46A1 surface (29). Notably, EFV is a pleiotropic small molecule with known off-target actions (e.g., GABA-A receptor modulation, effects on mitochondrial function) along with CYP46A1 activation (30, 31). In OIR mice treated with 20 mg/kg EFV, a dose derived from prior studies of its use therapeutically (32), from P7-P17 (both hyperoxia and hypoxia phases), the size of the avascular area and extent of pathological neovascularization were significantly reduced (Figures 3A–D). The AngioTool-derived parameters provide quantitative insights into retinal vascular architecture and its pathological alterations. Total vessel length reflects the extent of vascular coverage; a reduction in OIR mice indicates vaso-obliteration and impaired physiological vascularization (Figures 3E–G). The number of vessel junctions represents branching complexity, which is essential for efficient perfusion; its decrease in OIR retinas signifies disrupted vascular network integrity. Conversely, an increased number of vessel endpoints suggests excessive sprouting and incomplete vessel connections, characteristic of pathological neovascularization. Improvement in vessel length and junctions, along with reduced endpoints in EFV-treated OIR mice (Figures 3E–G), indicates restoration toward a more organized and physiologically functional vascular network, highlighting the therapeutic potential of EFV in normalizing angiogenesis.

To better understand the effect of EFV treatment on CYP46A1 activity in the retina, we analyzed 24HC levels in RA, OIR, and EFV-treated OIR mice retinas using an ELISA assay. As shown in Figure 3H, levels of 24HC were significantly improved in EFV-treated mice retinas compared to OIR samples, providing evidence for increased CYP46A1 activity. Collectively, these data provide evidence in support of the positive influence of maintaining CYP46A1 activation against the development of OIR pathology in mice and potentially ROP in humans.

**Figure 4:**
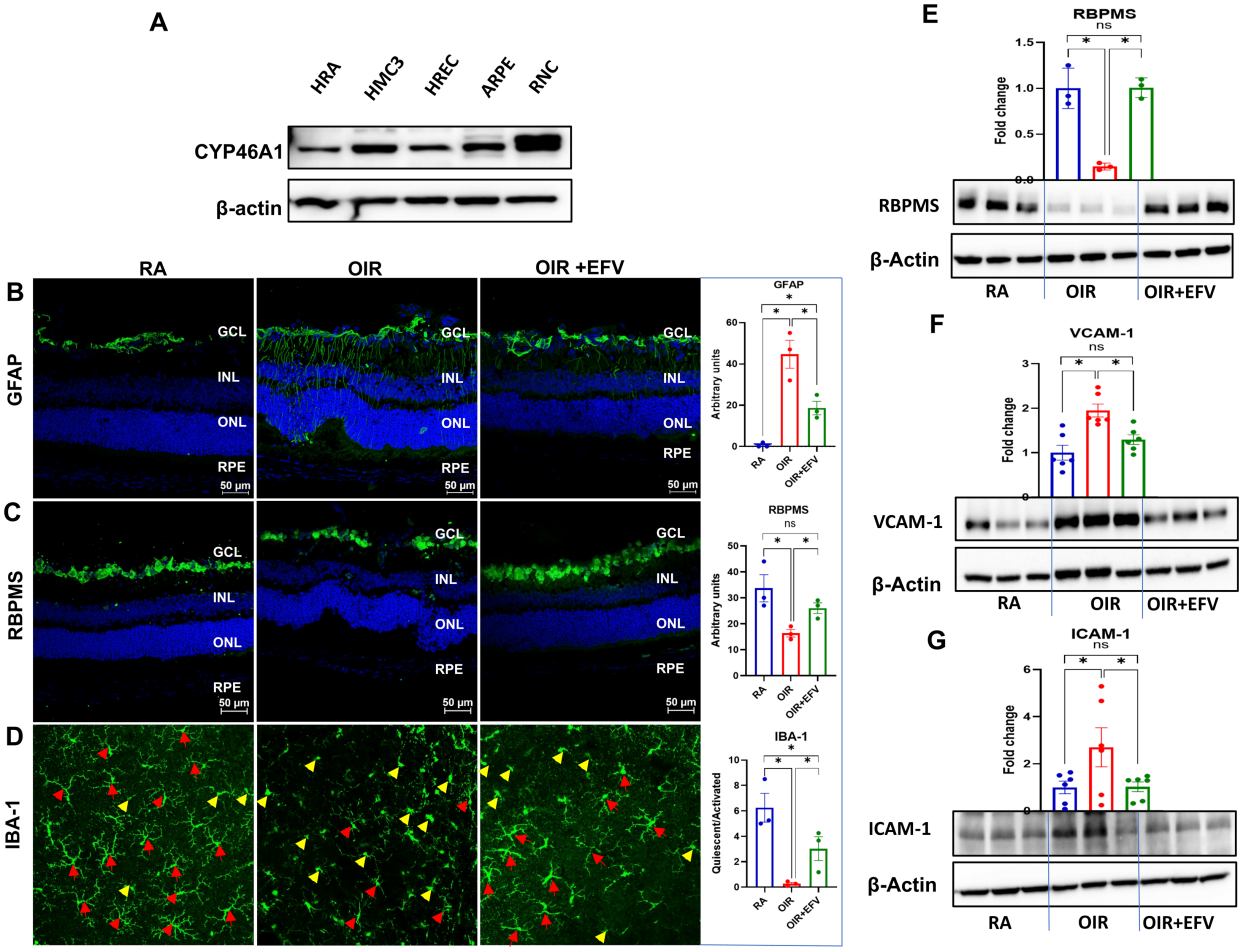
**(A)** Western blot analyses were performed using protein isolated from human retinal astrocytes (HRA), human brain microglia (HMC3), human retinal endothelial cells (HREC), human retinal pigment epithelial cells (ARPE), and differentiated retinal neuronal cells (RNC). Immunofluorescence staining of retinal cross sections from each experimental group to evaluate **(B)** GFAP and **(C)** RBPMS expression at P17. **(D)** IBA-1 immunostaining in representative retinal flat mount images from P17 mice of each group to identify quiescent (red arrowheads) or activated (yellow arrowheads) retinal microglia. **(E–G)** Western blot analyses of GFAP, RBPMS, VCAM-1, and ICAM-1 expression. β-Actin was used as a loading control. Representative data presented in this figure are derived from 3–6 retinas per group, each obtained from a different mouse.

Immunofluorescence analyses revealed diffuse CYP46A1 expression throughout the developing inner retina (Supplementary Figures 1, 2). To better understand the cell-type-specific expression of CYP46A1, Western blot analyses were performed using protein isolated from human retinal astrocytes (HRA), human brain microglia (HMC3), human retinal endothelial cells (HREC), human retinal pigment epithelial cells (ARPE), and differentiated retinal neuronal cells (RNC), which are all key in OIR pathology (33) (Figure 4A). CYP46A1 expression was detected in each cell type. To better understand the mechanisms responsible for the improvements in OIR pathology realized in the presence of EFV/increased CYP46A1 expression atop the impact on retinal endothelial cells/blood vessels, we further investigated glial responses in the retina by examining macroglial (astrocytic and Müller glial) reactivity and microglial activation, as well as retinal ganglion cell viability. GFAP upregulation is a well-established indicator of reactive gliosis in retinal macroglia (34); however, GFAP alone cannot distinguish astrocytes from Müller glia, as both cell types increase GFAP expression under stress or injury. Consistent with prior reports of gliosis in OIR (35, 36), we observed increased GFAP immunoreactivity in the OIR group (Figure 4B). EFV treatment attenuated this gliotic response, as evidenced by reduced GFAP signal intensity in EFV-treated OIR retinas. Because GFAP does not exclusively label Müller cells, these findings should be interpreted as reflecting general macroglial reactivity, rather than definitive evidence of Müller-cell–specific activation. Retinal ganglion cell survival was evaluated using the RGC-specific marker RBPMS (37), which demonstrated that EFV treatment preserved RGC viability in OIR mice (Figure 4C).

Microglia directly guide vascular growth by interacting with the filopodia of endothelial tip cells during retinal vasculature formation (38). Notably, in ischemic retinas, microglia tend to aggregate in ischemic and neovascularization regions, which are implicated as key contributors to the pathological processes. Infiltrating immune cells and local inflammatory signals can activate microglia. Activated microglia, in turn, produce cytokines and chemokines that further upregulate VCAM-1 and ICAM-1 expression, creating a vicious cycle of inflammation (39). The inflammatory milieu created by activated microglia and infiltrating immune cells can disrupt the normal angiogenic process. This disruption can lead to the aberrant growth of retinal blood vessels characteristic of the later stages of ROP. Retinal flat mounts from RA, OIR, and OIR + EFV treated animals were subjected to immunofluorescence analyses of IBA-1 to facilitate the identification of ramified (quiescent) and amoeboid (activated) microglia (Figure 4D). Ramified microglia were predominant in RA retinal flat mounts; however, flat mounts prepared from OIR mice were riddled with amoeboid microglia. Congruent with observations above, EFV treatment significantly reduced microglia activation, which is associated with reduced inflammation in OIR mouse retinas (Figure 4D). Western blot analyses confirm immunofluorescence staining results wherein EFV treatment significantly limited inflammation, ameliorated reactive gliosis, and prevented neuronal cell loss in EFV-treated OIR mice (Figures 4E–G).

Collectively, while EFV treatment improved retinal vascular outcomes in our OIR model and coincided with increased CYP46A1 immunoreactivity/activity and elevated 24HC, these findings demonstrate association rather than causation with respect to specific targeting of CYP46A1. Further studies designed to directly determine target dependence will benefit from testing EFV in a retina-specific CYP46A1 conditional knockout model. This way, EFV-protective mechanisms could be examined to see whether protective effects need CYP46A1 or if other mechanisms have led to the observed outcome. Although these experiments are beyond the scope of the present work. We hope that the application of conditional genetic tools in combination with additional pharmacologic interventions (e.g., EFV metabolites, alternate CYP46A1 modulators) will help to elucidate the degree to which CYP46A1 is involved in the effects of EFV in future studies.

Repurposing EFV to use in preterm infants comes with translational limitations as well. EFV is known to have significant neuropsychiatric and hepatic adverse effects in humans (40–42), and its safety profile has not been established in neonatal or premature populations. So while our results indicate EFV-associated enhancements of retinal sterol metabolism and vascular pathology, these findings should not be interpreted as direct support for EFV as a therapeutic option for infants. Instead, they highlight the wider scope of targeting sterol-regulating pathways and warrant the further development of safer, retina-specific CYP46A1 modulators with an improved safety margin for vulnerable neonatal populations.

## 4 Conclusion

The cytochrome P450 enzyme CYP46A1 is a major factor mediating cholesterol turnover in the retina, and aberrant CYP46A1 activity in the adult eye has been related to impaired cholesterol homeostasis and retinal dysfunction. In this article, we provide the first evidence, to our knowledge, that CYP46A1 expression is developmentally regulated in the neonatal retina and that with EFV therapy, the relevant pathological endpoints of OIR are ameliorated in association with increased CYP46A1 activity and elevated retinal 24HC levels. Nevertheless, we do not yet fully understand the precise mechanism(s) underlying these EFV-related benefits. As EFV is pleiotropic and our analysis did not include downstream pathway analyses or genetic loss-of-function models, it is not feasible to determine whether these effects are due to enhanced cholesterol elimination, 24HC–LXR signaling, bile acid-related pathways, or other EFV-responsive cascades. Thus, caution should be exercised with regard to the detailed mechanistic basis of these improvements. Precise pathway resolution will need to depend on focused studies of canonical LXR genes, bile-acid profiles, and, eventually, retina-specific Cyp46a1 conditional knockout models that are not documented in this brief research report, but are ongoing efforts to identify this phenomenon. Taken together, these findings offer preliminary consideration for modulation of retinal sterol metabolism and suggest its potential for OIR pathology amelioration as support for mechanistic investigations to elucidate how the pathways associated with CYP46A1 are involved in vascular protection in the developing retina.

## Data Availability

The RNA Seqdata presented in the study are deposited in Figshare (https://doi.org/10.6084/m9.figshare.31320685) The original contributions presented in the study are included in the article/Supplementary material, further inquiries can be directed to the corresponding author.

## Footnotes

1
http://oirseg.org
