# Supplementary material for: Efavirenz treatment improves retinal vaso-obliteration and pathological neovascularization in a mouse model of retinopathy of prematurity
Source: Front Med (Lausanne). 2026 Feb 23;13:1745351. doi: 10.3389/fmed.2026.1745351 (PMC12967956; doi:10.3389/fmed.2026.1745351)
Supplement: Supplementary file 1 [file Data_Sheet_1.pdf]

Efavirenz Treatment Improves Retinal Vaso-Obliteration and Pathological Neovascularization in a Mouse Model of Retinopathy of Prematurity

Briah Bailey[1#], Josephine Rudd Zhong Manis[2#], Gayatri Seth[1], Shubhra Rajpurohit[3], Allston Oxenrider[2], Pamela M. Martin[1], Ravirajsinh N. Jadeja[1], Menaka C. Thounaojam[1,2,3*]

[1]Department of Biomedical Sciences, School of Graduate Studies, Meharry Medical College, Nashville, TN, USA [2]Departments of Cellular Biology and Anatomy and [3]Ophthalmology, Medical College of Georgia at Augusta University, Augusta, GA, USA

#Equal contribution.

*Correspondence: menaka.thounaojam@mmc.edu; Tel.: (+1 6153275940)

Supplementary Data:

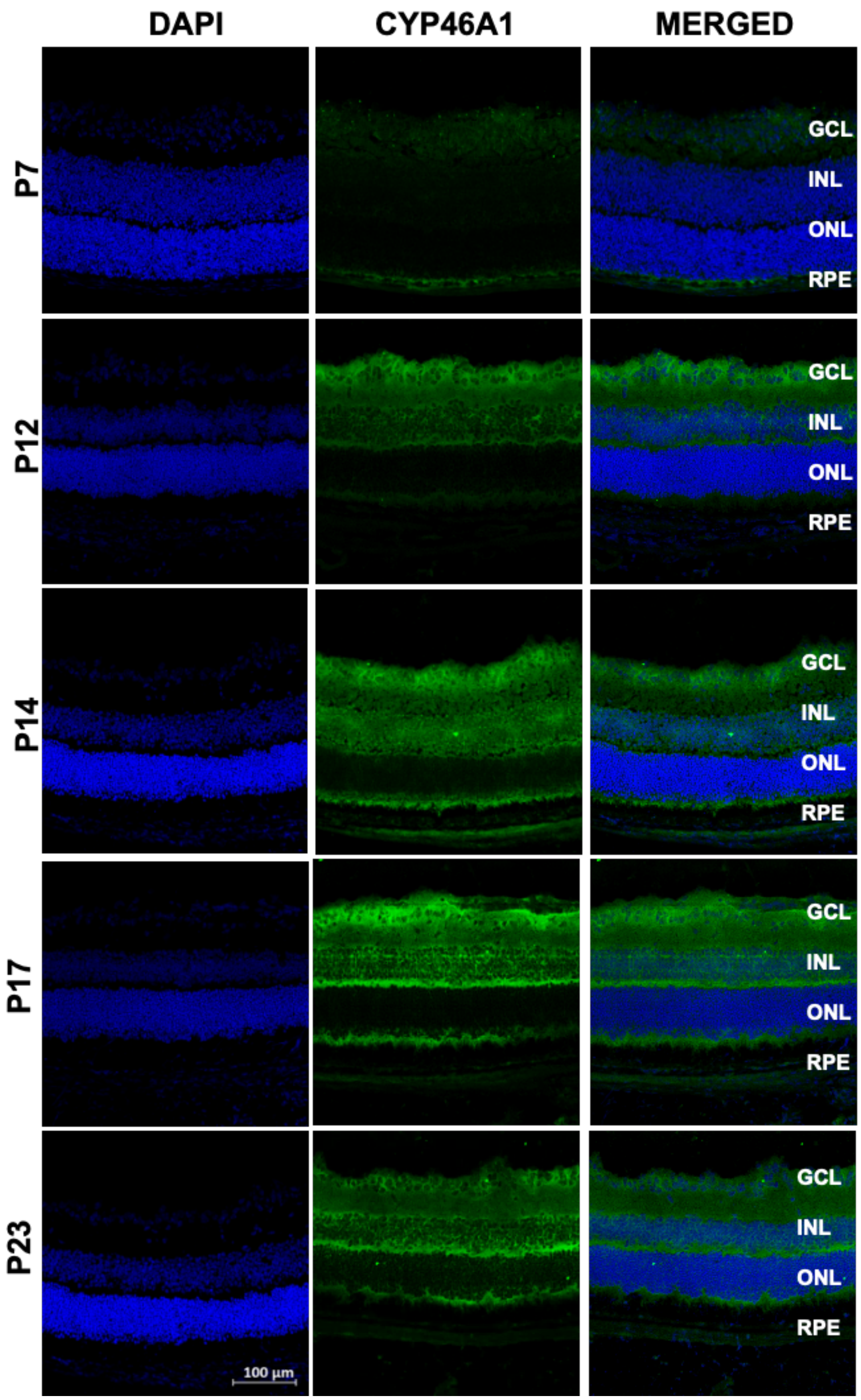

Supplementary Figure 1. Immunofluorescence analysis of retinal cross-sections showing CYP46A1 expression across postnatal development. Representative confocal images of mouse retinal sections labeled with CYP46A1 (green) and counterstained with DAPI (blue). Retinal layers are indicated on the right: ganglion cell layer (GCL), inner nuclear layer (INL), outer nuclear layer (ONL), and retinal pigment epithelium (RPE).

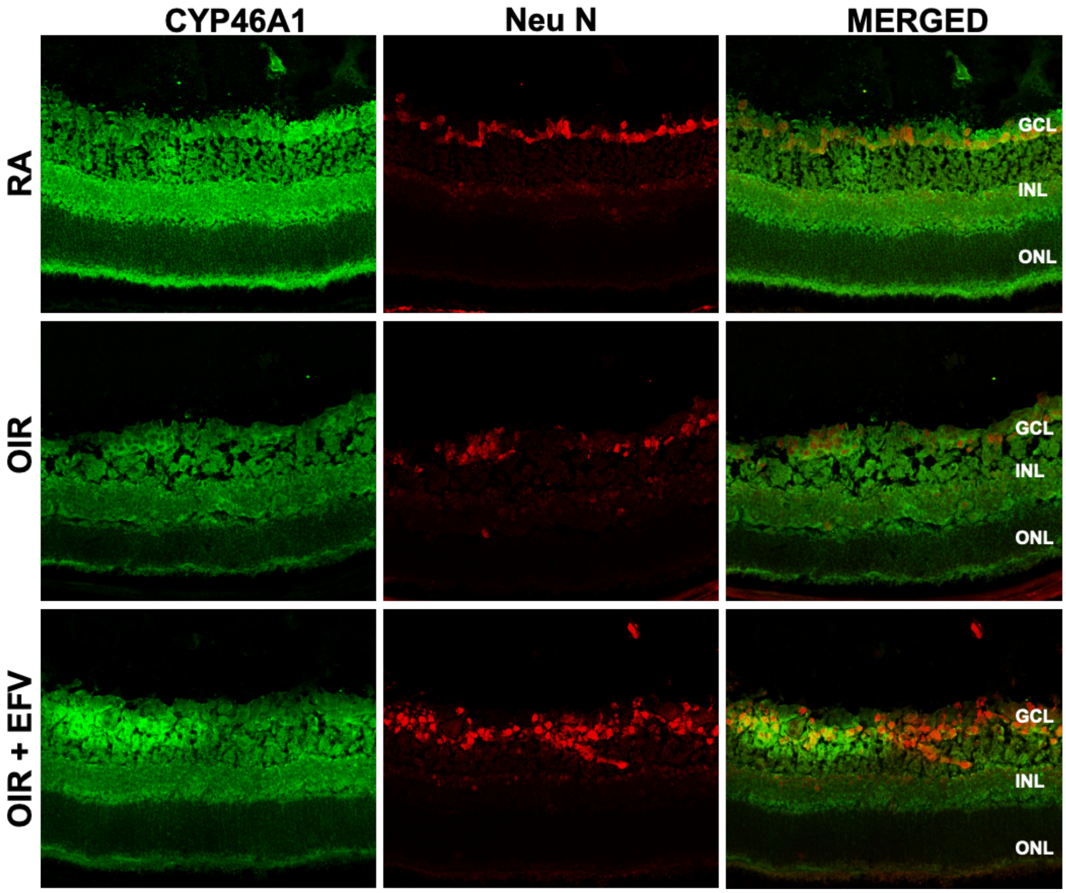

Supplementary Figure 2. Immunofluorescence colocalization of CYP46A1 and NeuN in mouse retinal cross-sections. Representative confocal images of mouse retinal sections stained for CYP46A1 (green) and the neuronal marker NeuN (red). Left panels show CYP46A1 immunoreactivity, middle panels show NeuN labeling, and right panels display merged images demonstrating regions of colocalization (yellow). CYP46A1 expression is prominent in the inner retinal layers, with partial overlap with NeuN-positive neuronal cell bodies. NeuN labeling localizes primarily within the ganglion cell layer (GCL) and inner nuclear layer (INL). Retinal layers are indicated on the right: ganglion cell layer (GCL), inner nuclear layer (INL), and outer nuclear layer (ONL).
